# Blockade of VEGFR1 and 2 Suppresses Pathological Angiogenesis and Vascular Leakage in the Eye

**DOI:** 10.1371/journal.pone.0021411

**Published:** 2011-06-22

**Authors:** Hu Huang, Jikui Shen, Stanley A. Vinores

**Affiliations:** Wilmer Eye Institute, Johns Hopkins University School of Medicine, Baltimore, Maryland, United States of America; Washington University School of Medicine, United States of America

## Abstract

**Objective:**

VEGFR1 and 2 signaling have both been increasingly shown to mediate complications of ischemic retinopathies, including retinopathy of prematurity (ROP), age-related macular degeneration (AMD), and diabetic retinopathy (DR). This study evaluates the effects of blocking VEGFR1 and 2 on pathological angiogenesis and vascular leakage in ischemic retinopathy in a model of ROP and in choroidal neovascularization (CNV) in a model of AMD.

**Materials and Methods:**

Neutralizing antibodies specific for mouse VEGFR1 (MF1) and VEGFR2 (DC101) were administrated systemically. CNV was induced by laser photocoagulation and assessed 14d after laser treatment. Retinal NV was generated in oxygen-induced ischemic retinopathy (OIR) and assessed at p17. NV quantification was determined by measuring NV tufts and vascular leakage was quantified by measuring [^3^H]-mannitol leakage from blood vessels into the retina. Gene expression was measured by real-time quantitative (Q)PCR.

**Results:**

VEGFR1 and VEGFR2 expressions were up-regulated during CNV pathogenesis. Both MF1 and DC101 significantly suppressed CNV at 50 mg/kg: DC101 suppressed CNV by 73±5% (p<0.0001) and MF1 by 64±6% (p = 0.0002) in a dosage-dependent manner. The combination of MF1 and DC101 enhanced the inhibitory efficacy and resulted in an accumulation of retinal microglia at the CNV lesion. Similarly, both MF1 and DC101 significantly suppressed retinal NV in OIR at 50 mg/kg: DC101 suppressed retinal NV by 54±8% (p = 0.013) and MF1 by 50±7% (p<0.0002). MF1 was even more effective at inhibiting ischemia-induced BRB breakdown than DC101: the retina/lung leakage ratio for MF1 was reduced by 73±24%, p = 0.001 and for DC101 by 12±4%, p = 0.003. The retina/renal leakage ratio for MF1 was reduced by 52±28%, p = 0.009 and for DC101 by 13±4%, p = 0.001.

**Conclusion:**

Our study provides further evidence that both VEGFR1 and 2 mediate pathological angiogenesis and vascular leakage in these models of ocular disease and suggests that antagonist antibodies to these receptor tyrosine kinases (RTKs) are potential therapeutic agents.

## Introduction

Pathological angiogenesis/neovascularization (NV) and vascular leakage/permeability due to blood-retinal barrier (BRB) breakdown are the two major sight-limiting complications in ROP, DR, and AMD. The mechanisms by which pathological angiogenesis and BRB dysfunction develop in these ischemic retinopathies have been investigated extensively and a number of target molecules that stimulate the vascular complications due to the ischemia or diabetes and agents that can suppress the pathological processes have been identified and characterized. Among them, VEGF has been identified as a key angiogenic and vasopermeability factor that is up-regulated in ischemic retinopathies, such as ROP, AMD, and DR, where it can promote BRB breakdown and NV [1]–[6]. Even relatively minor states of hypoxia can result in the induction of VEGF [7]–[10] through a family of hypoxia-inducible transcription factors (HIFs) that bind to a hypoxia response element (HRE) in the *VEGF* promoter [10]. Using mice with a deletion of the HRE of the *VEGF* promoter, which renders them incapable of up-regulating VEGF in response to HIF, there was almost a total inhibition of retinal NV and vascular leakage due to BRB breakdown in a model of OIR and of CNV in a model of AMD [11], showing that these activities are mediated through HIF-induced VEGF in these models. In the eye, VEGF can be expressed by multiple cell types including Müller cells, retinal pigment epithelium (RPE), endothelial cells, glial cells, ganglion cells and photoreceptors, and its mutation or over-expression specifically in certain cell types is desired to investigate the role of VEGF from different cell sources. For instance, with the conditional knockout tool Cre/LoxP system, VEGF was mutated specifically in Müller cells, leading to dramatic suppression of retinal NV, inflammation, and vascular leakage due to BRB breakdown in ischemia and/or diabetes [12]. In contrast, VEGF over-expression in certain cells can lead to pathological consequences. One example is V6 VEGF transgenic mice, which over-express VEGF in the photoreceptors under control of the rhodopsin promoter, which leads to increased retinal NV and BRB breakdown [13]. In V6 mice, the outer retina is primarily affected, but if the source of VEGF is in the inner retina, such as astrocytes, Müller cells, or ganglion cells, the inner retina is primarily affected, showing that the source of VEGF is important, as well as its levels and time of expression [14].

The development of antagonists, chemical compounds, or other small molecules (i.e., small interfering (si)RNA) to neutralize VEGF has dramatically advanced the field of anti-angiogenic therapy and anti-VEGF therapy has now become widely used to treat angiogenesis-dependent disorders such as cancer and retinopathies like neovascular AMD [15], [16]. Despite the clinical benefits, some challenges exist for anti-angiogenic therapy, which was described in detail in the literature [17]–[21]. Briefly, they include (i) half of patients don't respond to anti-VEGF therapy due to the existence of other angiogenic factors, (ii) drug resistance to anti-VEGF therapy as a result of the selective up-regulation of other angiogenic factors and/or prevention of drug from arriving at the targeted endothelial cell sites due to surrounding pericytes and/or extracellular matrix, (iii) repetitive delivery of anti-VEGF agent is often needed to effectively suppress neo-vessels due to their failure to regress and/or recurrence of these abnormal vasculatures, and (iv) VEGF is also a survival factor for vascular endothelial cells and neurons and adverse side effects have been occasionally reported for anti-VEGF therapy. Additionally, the multiple VEGF isoforms resulting from alternative splicing and the complexity and interaction of its receptor signaling systems provide a new dimensional challenge to manage anti-VEGF therapy.

VEGFR1 and 2 share high sequence homology, are both tyrosine kinase receptors (TKRs) and transmit signaling from several members of the VEGF family. VEGF-A is the common ligand for both receptors, but PlGF and VEGF-B are specific for VEGFR1 and VEGF-E is specific for VEGFR2 [20]–[24]. It has been long believed that VEGFR2 is the primary receptor by which VEGF mediates its permeability and angiogenic activities and that VEGFR1 is less potent and may act as a negative regulator for VEGFR2, especially during embryonic angiogenesis [25]. However, the accumulating evidence suggests that VEGFR1 signaling plays an important role in angiogenesis as well, particularly in pathological conditions [26], [27]. An antibody to PlGF, a ligand of VEGFR1, but not VEGFR2, is highly effective at suppressing tumor angiogenesis and choroidal neovascularization (CNV) in an experimental model of AMD and its efficacy is enhanced when used in combination with an antibody to VEGFR2, providing evidence that both receptors play a role [28]. Targeting VEGFRs, particularly VEGFR2, has provided clinical benefits for patients suffering from angiogenesis-dependent disorders, such as cancer, but this treatment has been confined to patients with cancer and is not yet available for the treatment of eye diseases (http://www.angio.org/ua.php). Anti-PlGF, however, is in clinical trials for cancer and ocular disease. Direct signal transduction and synergistic interactions with VEGFR2 are involved in the mechanisms by which VEGFR1 regulates pathological angiogenesis and vascular permeability [28]. Further cross-talk between VEGFR1 and 2 adds to the complexity. The present study was conducted to try to resolve some of the confusion about the respective roles of VEGFR1 and 2 and give further insight into how they participate in pathological angiogenesis and vascular leakage by blocking VEGFR1 and/or 2 signaling with specific neutralizing antibodies to VEGFR1 and 2 in laser-induced CNV and hypoxia-induced retinal NV models. The data from the present study is of significance to help design better therapeutic strategies for targeting the two TKRs to treat ischemic retinopathies.

## Methods

### Antagonist antibodies & systemic administration

MF1 and DC101 were provided by ImClone System (New York, NY), a wholly owned subsidiary of Eli Lilly and Company. The targets and specificity of MF1 have been described [27], as have those for DC101 in vitro [29] and in vivo [30]. Intraperitoneal (i.p.) injections of 12.5 mg/kg, 25 mg/kg, 50 mg/kg MF1, 50 mg/kg DC101, or 25 mg/kg MF1+25 mg/kg DC101 in PBS were administered for both CNV and ROP models. An equal volume of PBS or an equal concentration of non-specific rat antibody in PBS were used as controls and the results of both controls were identical. The doses were based on the results of other studies [28], [31]–[33]. For the timing in CNV, the mice were treated right after laser treatment and followed by every other day (7 treatments in total), and for that in OIR, the mice were treated on P12 (immediately after the mice are removed from hyperoxic conditions) and on P15 (when the mice are in normal air for 3 days) (2 treatments in total).

### Mice

Animal use was in accordance with the approved protocols by the Institutional Animal Care and Use Committee of Johns Hopkins University School of Medicine and the guidelines of the Association for Vision and Ophthalmology. The 6–8-week old and 15 or 16 day pregnant C57BL/6 mice were purchased from Charles River (Wilmington, MA) and housed at the Wilmer Woods Animal Facility of Johns Hopkins University.

### Real-time quantitative (Q) PCR

Real-time QPCR was performed in 96-well plates with the Bio-Rad IQ5 system, as described previously with some modifications [34], [35]. Each 20 µl of reaction contained 10 µl of 2×SYBR Green Supermix, 20 nM of target gene primer mix, and 20–50 ng of a cDNA template. QPCR conditions included an initial denaturing step for 3 min at 95°C, followed by 40 cycles (95°C for 15 sec, 58°C for 20 sec, and 72°C for 25 sec). Primers were optimized by melting curve profiles and agarose gel analysis. The quantification was calculated using comparative threshold cycles (*C*_T_): the data were normalized by subtracting the difference of the threshold cycles (*C*_T_) between the gene of interest's *C*_T_ and the housekeeping gene Cycophilin's *C*_T_ (Δ*C*_T_ = gene of interest *C*_T_-Cycophilin *C*_T_) for each sample. The Δ*C*_T_ difference between two samples was designated as ΔΔ*C*_T_ (ΔΔ*C*_T_ = sample1 Δ*C*_T_− sample2 Δ*C*_T_). The fold change of the two samples was calculated as 2^−ΔΔ*C*T^. The sequences of primers were cited from published literature: cycophilin, VEGF, PlGF, VEGFR1 and 2 were obtained from Robinson, et al. [36]. VEGF-B was obtained from Zhong, et al. [37]. SDF-1, CXCR-4, Ang2, Tie2, CD117, SCF, Epo, and EpoR were obtained from Yoshida, et al. [38]. For statistical analysis, 3 replicates from 5 animals were performed on each treatment or PBS control group (PBS acts as control because non-specific antibodies were used in the pilot studies and were found to be show the same results as PBS).

### Mouse model of choroidal neovascularization (CNV)

CNV was induced by laser photocoagulation-induced rupture of Bruch's membrane [39]. C57BL/6J (6–8 week-old) mice were anesthetized with ketamine hydrochloride (100 mg/kg body weight) and xylazine (4 mg/kg body weight) and the pupils were dilated with 1% tropicamide. Laser photocoagulation (75 µm spot size, 0.1 sec duration, 120 mW) was performed in the 9, 12, and 3 o'clock positions of the posterior pole of the retina with the slit lamp delivery system of an Oculight GL diode laser (Iridex, Mountain View, CA) and a handheld cover slip as a contact lens to view the retina. Production of a bubble at the time of laser, which indicates rupture of Bruch's membrane, is an important factor in obtaining CNV. Therefore, only burns in which a bubble was produced were included in the study. Two weeks after rupture of Bruch's membrane, anesthetized mice were perfused with 50 mg/ml fluorescein-labelled-dextran (2×10^6^ average molecular weight, Sigma-Aldrich, St. Louis, MO). The eyes were then dissected and fixed in 10% buffered formalin for 3 hours and choroidal flat mounts were prepared. Choroidal flat-mounts were washed with 0.01 M PBS (pH 7.4) and blocked with 4% normal goat serum without addition of detergent (i.e., 0.1% Triton X-100) for increased permeabilization. They were incubated overnight at 4°C with FITC- or Alexa Fluor 594-conjugated isolectin and polyclonal antibodies against CD31 or polyclonal antibodies against CD45 (1∶100; Cell Signaling Technology, Boston, MA) or Pacific Blue-conjugated rat monoclonal antibody against mouse F4/80 (1∶100; Invitrogen, Carlsbad, CA ). Negative control sections were similarly treated, but the primary antibodies were omitted. Specimens were rinsed and incubated for 1 hr with Alexa Fluor 594 or 488-conjugated goat anti-rabbit IgG (1∶1000; Invitrogen). Fluorescent microphotography was performed on a Zeiss Axioplan2 epifluorescence microscope. Image-Pro Plus software (Media Cybernetics, Silver Spring, MD) was used to measure the total area of CNV at each rupture site by personnel blinded as to study treatments and groups and the numbers of positive cells were counted and representative images were photographed. The known cell types that can be labeled with each of the markers are shown in Table 1.

**Table 1:** Known cell types that can be labeled with each of the markers.

Staining Methods	Cell Types	References
Lectin	Vascular endothelial cells, macrophages, microglia	[51], [52]
CD31	Vascular endothelial cells	[42]
CD45	Leukocytes (i.e. macrophages), microglia	[68]
F4/80	Macrophages, microglia	[27]

### Mouse model of oxygen-induced retinal neovascularization (NV)

The oxygen-induced ischemic retinopathy (OIR) model was produced in C57BL/6 mice according to the method previously described [40]. In brief, litters of 7-day old (P7) mice were exposed to an atmosphere of 75% oxygen in an airtight incubator for 5 days (P12), after which they were returned to room air for 5 days (P17). Quantification of retinal NV was carried out as described previously [41], [42]. For quantification of OIR-induced retinal neovascularization, P17 mice were given an intraocular injection of 1 µl of rat anti-mouse platelet endothelial cell adhesion molecule-1 (PECAM-1) antibody (Pharmingen, San Jose, CA) under a dissecting microscope with a Harvard pump microinjection apparatus. Mice were euthanized 12 hours after injection and eyes were fixed in PBS-buffered formalin for 5 hours. Retinas were dissected, washed and incubated with goat anti-rat polyclonal antibody conjugated with Alexa Fluor 488 or were labeled with griffonia simplicifolia-594 (Invitrogen, Carlsbad, CA) for 45 min. Images of each of the 4 quadrants of whole-mounted retina were taken at 5x magnification on a Zeiss Axioplan 2 microscope and imported into Adobe Photoshop. Retinal segments were merged to produce an image of the entire retina. Neovascular tuft formation was quantified on retinal flat mounts with fluorescence microscopy using Image Pro Plus software.

### BRB assay

The quantitative BRB assay was performed according to a previously described technique [43]. Mice were sedated as above and given an i.p. injection of 1 µCi/gram body weight of [^3^H]mannitol. One hr after injection, the mice were sedated and retinas from experimental and control eyes were rapidly removed. The posterior portion of the globe was firmly grasped with forceps and a razor blade was used to cut across the cornea and extrude the lens, vitreous, and retina. Retinas were dissected free from lens, vitreous, and any RPE that was extruded, and were placed within pre-weighed scintillation vials within 30 seconds of sacrifice. The thoracic cavity was opened and the left superior lobe of the lung was removed, blotted free of excess blood, and placed in another pre-weighed scintillation vial. A left dorsal incision was made and the retroperitoneal space was entered without entering the peritoneal cavity. The renal vessels were clamped with a forceps and the left kidney was removed, cleaned of fat, blotted, and placed into a pre-weighed scintillation vial. Superficial liquid was allowed to evaporate over 20 min from the open vials. The vials containing the tissue were weighed and tissue weights were calculated and recorded. One ml of NCSII solubilizing solution was added to each vial and the vials were incubated overnight in a 50°C water bath. Solubilized tissue was brought to room temperature (RT) and decolorized with 20% benzoyl peroxide in toluene in a 50°C water bath. The vials were brought to RT and 5 ml of Cytoscint ES and 30 µl of glacial acetic acid were added. The vials were stored for several hours in darkness at 4°C to eliminate chemoluminescence. Radioactivity was counted with a LS 6500 Liquid Scintillation Counter (Beckman, Brea, CA). The CPM/mg tissue was measured for lung, kidney, and experimental and control retina. Retina/lung, retina/kidney, and lung/kidney ratios were calculated and compared.

### Statistical Analysis

Statistical comparisons were made using analysis of variance (ANOVA) or a linear mixed model [44]. P-values for comparison of treatments were adjusted for multiple comparisons using Dunnett's method. For data sets with two groups, statistical analyses were performed by the unpaired *t*-test for Excel 2003 (Microsoft, Redmond, WA).

## Results

### Expression of VEGFR1 and 2 was up-regulated during CNV pathogenesis (Fig. 1)

**Figure 1:**
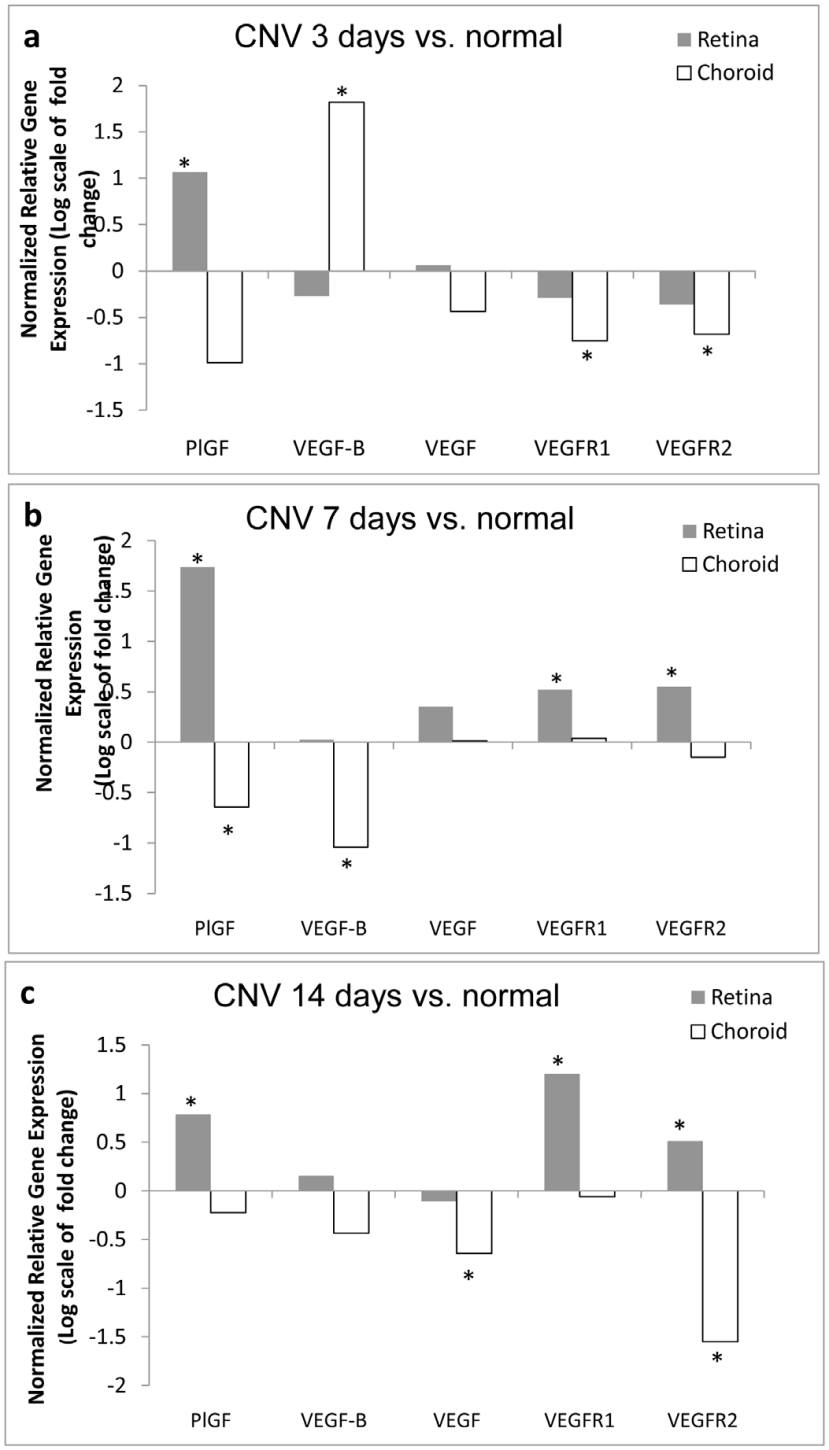
VEGFR1 and 2 and their ligands were up-regulated during CNV pathogenesis. Ten-twelve CNV lesions were created in one eye of each mouse for gene expression analysis and the fellow eye, which was not treated, served as a control. The results were expressed as normalized relative gene expression or the log(2) scale of the mean change fold over control from 5 mice. (a) Normalized relative gene expression over control for retinas with CNV lesions, 3 day after lasering; (b) Normalized relative gene expression over control for retinas with CNV lesions, 7 day after lasering; (c) Normalized relative gene expression over control for retinas with CNV lesions, 14 day after lasering. *: p<0.05 vs. control.

Gene expression of VEGFR1 and VEGFR2 and their ligands: VEGF, VEGFB, and PlGF, at mRNA level were examined at 3 days (early or initial stage), 7 days (intermediate or active stage) and 14 days (late or involution stage) after laser treatment. At 3 days after laser treatment, PlGF and VEGF-B showed significant up-regulations: PlGF had 2.1±0.7-fold higher expression in retina and VEGF had 3.9±1.4-fold higher expression in choroid than normal, No significant changes were detected for VEGF expression in retina, and the expression of VEGF receptors decreased slightly in retina and choroid compared to normal. At 7 days after laser treatment, PlGF showed the most pronounced increase: PlGF was 3.3±1.4-fold higher in laser-treated retina than normal. VEGF, VEGFR1 and VEGFR2 showed a slight increase in laser-treated retina, while PlGF and VEGF-B showed a slight, but significant decrease in choroid following laser photocoagulation. At 14 days, PlGF, VEGFR1 and VEGFR2 showed significant up-regulations in retina to some degrees: VEGF increased 1.7±0.5-fold, VEGFR1 increased 2.4±0.7-fold, and VEGFR2 increased 1.4±0.2-fold, but the expressions of VEGF and VEGFR2 were slightly decreased in choroid.

### Blockade of VEGFR1 and 2 suppressed CNV (Fig. 2)

**Figure 2:**
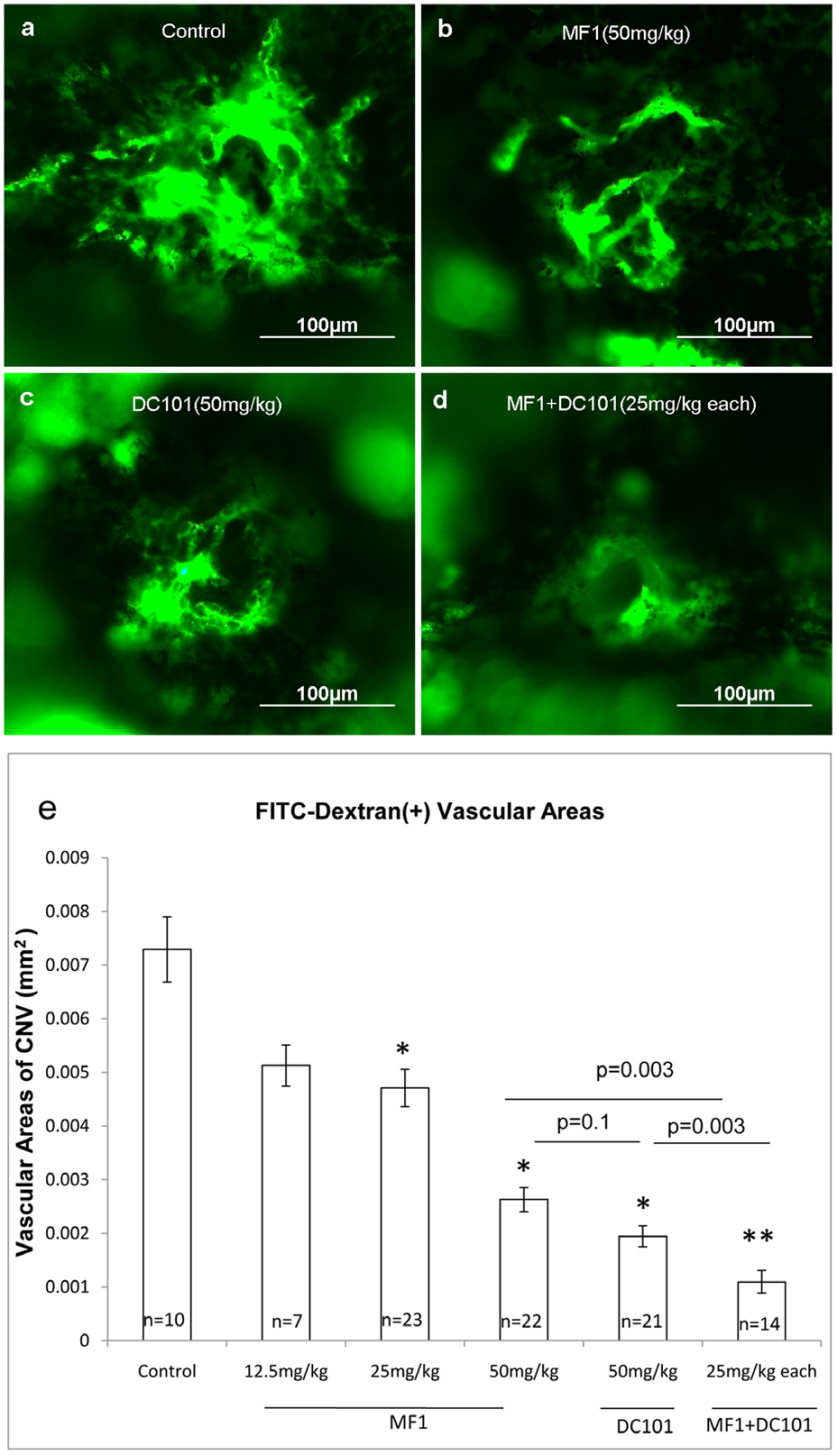
Blockade of VEGFR1 and 2 suppressed CNV. Laser-induced ruptures of Bruch's membrane were performed as described in Methods. Intraperitoneal injections of indicated doses of MF1 or DC101 or MF1+DC101 were administered immediately after laser treatment and then every other day after lasering until 14 days, when CNV was assessed. Mice were perfused with FITC-labeled dextran and choroidal flat mounts were prepared and examined by fluorescence microscopy. Compared to control eyes (a), those injected with 50 mg/kg MF1 (b) or 50 mg/kg DC101 (c) showed a significant reduction of CNV area. CNV area in eyes injected with 25 mg/kg MF1+25 mg/kg DC101 (d) was significantly reduced compared to that obtained from control mice or from mice treated with MF1 or DC101 alone. (e) The area of CNV at each rupture site was measured by image analysis. Results are expressed as mean areas (mm^2^) of CNV±SE for each group calculated from indicated number (n) of CNV lesions. *: p values were less than 0.05 vs control; **: p values are less than 0.0001 vs. control.

With the two neutralizing antibodies MF1 and DC101, we evaluated the effects of blocking VEGFR1 or 2 signaling alone or together on CNV formation. The dosages evaluated included MF1: 12.5 mg/kg, 25 mg/kg, and 50 mg/kg, DC101: 50 mg/kg, and MF1+DC101: 25 mg/kg each. Compared to controls, both MF1 and DC101 suppressed CNV formation and MF1 did so in a dose-dependent manner, as was previously reported for DC101 [28]. At the 50 mg/kg dose, the inhibitory efficacy is slightly, but not significantly higher for DC101 than MF1 (64±6% for MF1 and 73±5%for DC101, p = 0.1); a combination of MF1 and DC101 (25 mg/kg each) showed a greater inhibitory efficacy than 50 mg/kg MF1 or 50 mg/kg DC101 alone (85±4% inhibitory efficacy by combination treatment, p = 0.0003 vs. MF1 or DC101) (Fig. 2). Grossly visible side effects were not observed with either antibody, but tissue analysis was not performed.

### Blockade of VEGFR1 and 2 resulted in an accumulation of microglia at the CNV surface (Fig. 3, 4)

**Figure 3:**
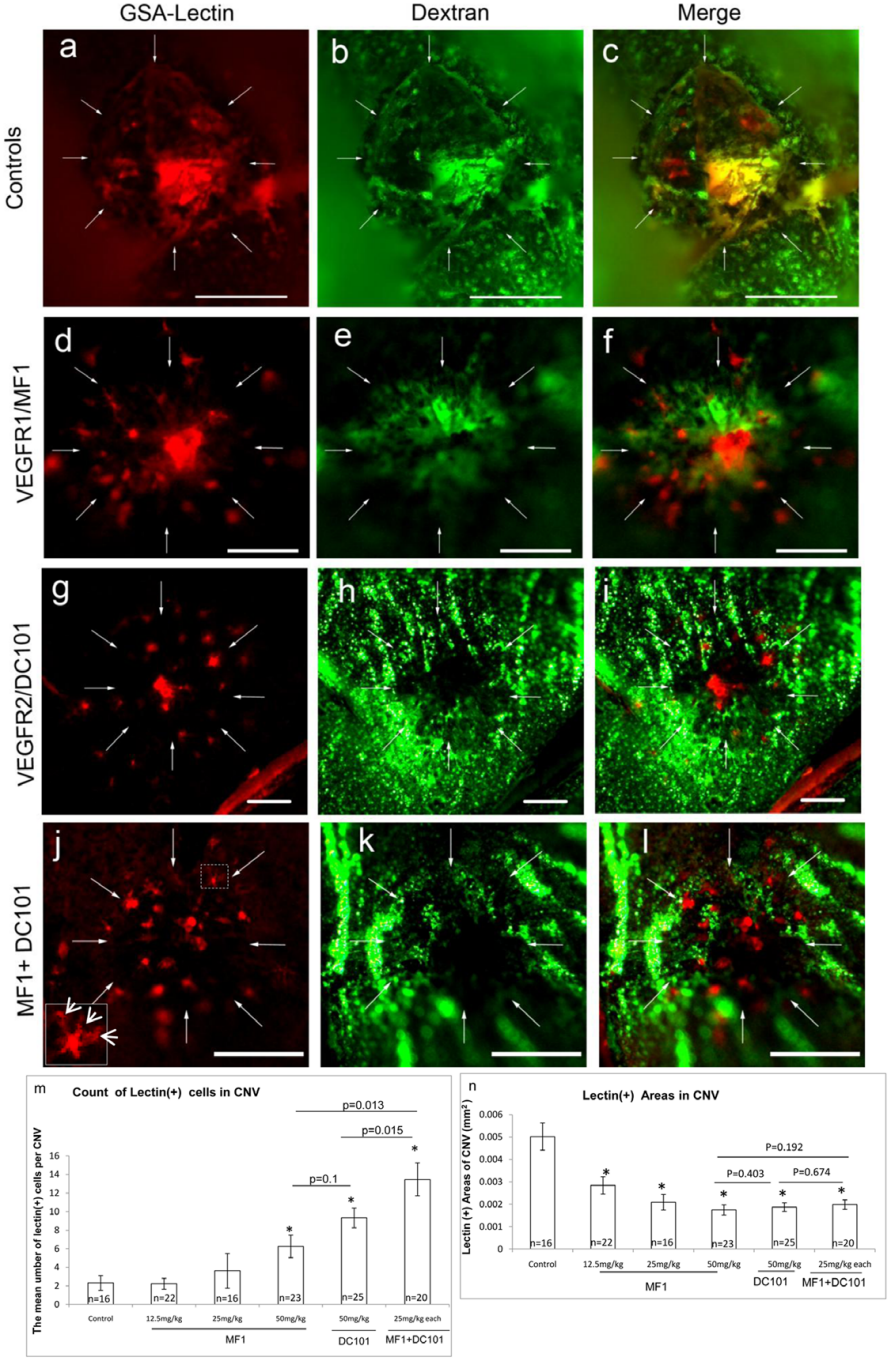
Blockade of VEGFR1 and 2 led to accumulation of Lectin^+^ cells at CNV surface. Representative CNV lesions, which were stained with GSA-isolectin (left column) and Fluorescein-Dextran (middle column), for controls (a–c), MF1 (d–f), DC101 (g–i) and MF1+ DC101 (j–l) are shown. The insert at the lower left of panel j is a higher magnification of the cell in the box, showing the morphological appearance of a microglial cell. The mean numbers of segregated lectin+ cells from each CNV lesion, which couldn't incorporate into vessel walls, are shown in panel m. Arrows define the limits of the CNV lesions. The quantification of lectin-positive area is shown in panel n. *: p values are less than 0.5 vs. control.

**Figure 4:**
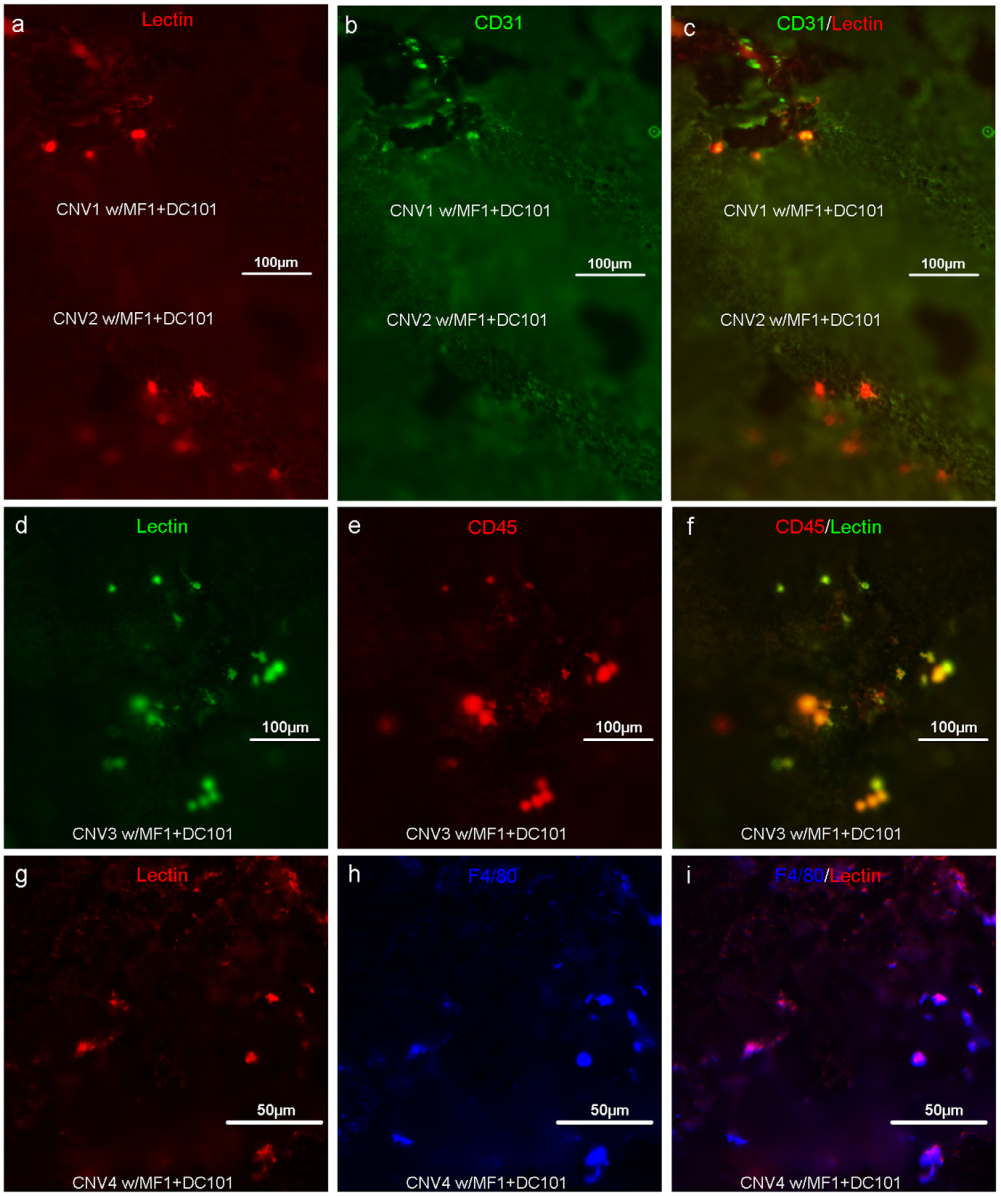
The Lectin^+^ cells at the CNV surface were identified as inflammatory cells. The choroidal flat mounts were prepared from the groups that were treated with MF1+DC101. CNV was double-labeled with lectin (a, d and g) and CD31, (b) CD45, (e) or F4/80 (h). The merged images are shown in c, f and i.

When we stained the CNV lesions with GSA-lectin, we observed that, unlike the controls, the lectin^+^ areas in CNV lesions from mice treated with MF1 and/or DC101 actually contained some segregated lectin^+^ cells or cell aggregates, which were not assembled or organized into integral vasculatures because the lectin^+^ areas showed mutually exclusive patterns when compared to the perfused FITC-Dextran^+^ areas (Fig. 3f, i & l), suggesting these cells or cell aggregates are either abnormal vasculatures without normal lumen structures, or non-vascular components, or both. The mean number of these segregated lectin^+^ cells from each CNV lesion significantly increased with combination treatments of MF1 and DC101, compared to single treatment groups (Fig. 3m).The further quantification of these lectin+ CNV areas (Fig. 3n), which is different from the staining of FITC-Dextran, representing all the perfused blood vessels in CNV, and from the results shown in Fig. 3n, which are only for those non-perfused components, reflects all the perfused and non-perfused vasculatures and imflammatory cells at the CNV surface, showed significant inhibition in all treatment groups compared to controls, but that of the combination of MF1 and DC101 did not show significant differences compared to single treatment alone, which may be due to the increased number of segregated or non-perfused lectin^+^ cells (Fig. 3m), which were further identified as retinal microglia at the CNV surface (see below).

Because we didn't include detergent when we immuno-stained the choroidal flat mounts, we expected only the cells at the superficial CNV area (on the retinal side), close to the sub-retinal space, the RPE, and the outer segments of the photoreceptors of the CNV lesions to be labeled. To identify the lectin^+^ cells at the CNV surface, a panel of antibodies, including CD31, CD45, and F4/80, were implemented. We first stained them with the endothelial cell marker, CD31, because we speculated they were vascular components, which had failed to assemble into neo vessels due to the lack of VEGFR1 and 2 signaling. However, we surprisingly found that these lectin^+^ cells had an undetectable to very weak signal for CD31 (Fig. 4a–c), but were positive for the leukocyte marker CD45 (Fig. 4d–f). Very often, these cells have multiple processes and microglia-like morphology (one example is shown in the insert of Fig. 3j). Further staining with F4/80 antibody defined them as residential microglia/macrophages (Fig. 4g–i). In contrast, the flat mounts of CNV from control groups were positive for CD31 but negative for CD45 (data not shown).

### Blockade of VEGFR1 and 2 reduced ischemia-induced retinal NV in OIR (Fig. 5 & 6)

**Figure 5:**
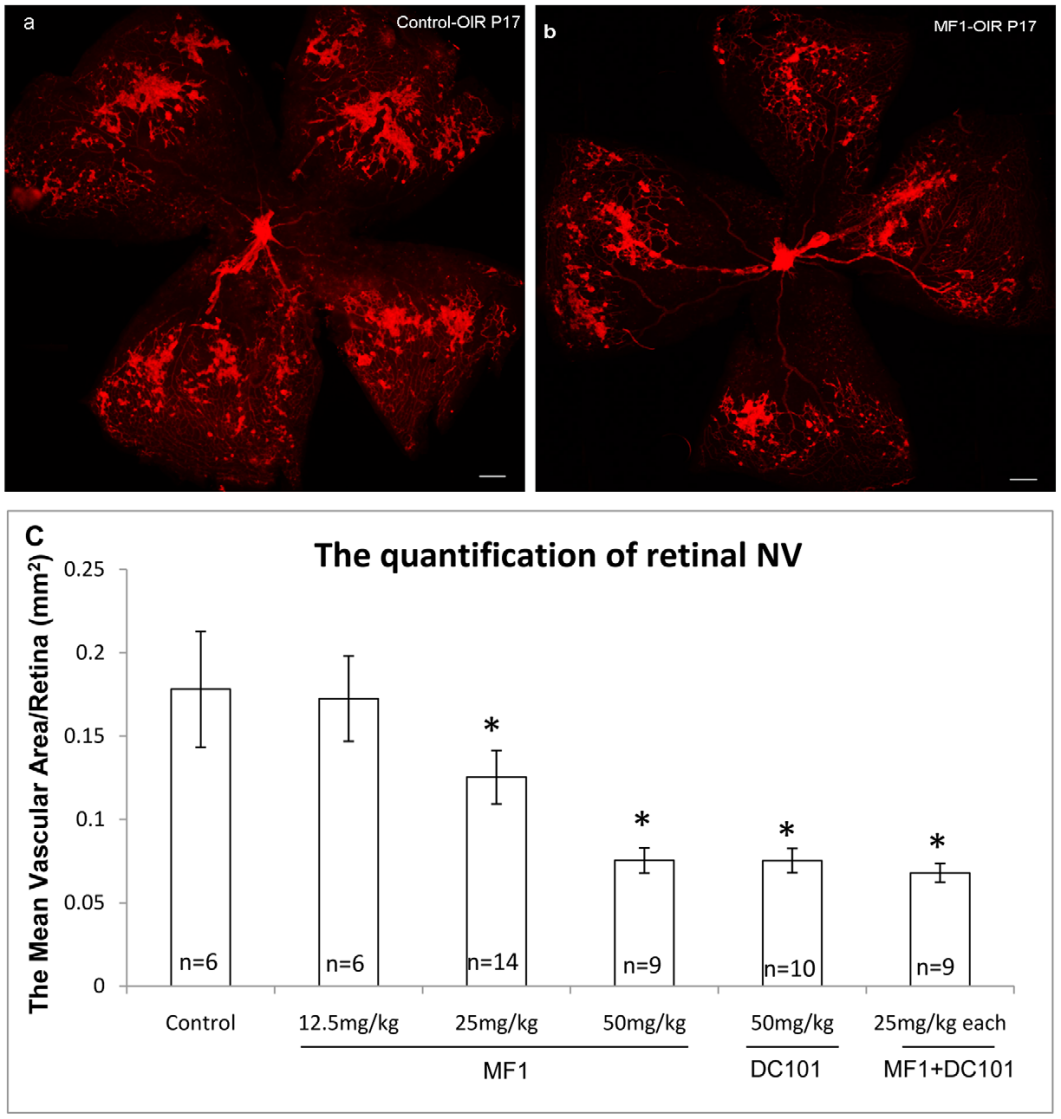
Blockade of VEGFR1 and 2 suppressed retinal NV in OIR. Neovascular tufts were labeled with griffonia simplicifolia lectin-594. Representative retinal NV for controls (a) and the combination treatment of MF1 and DC101 (b) were demonstrated. (c) The quantification of retinal NV.

**Figure 6:**
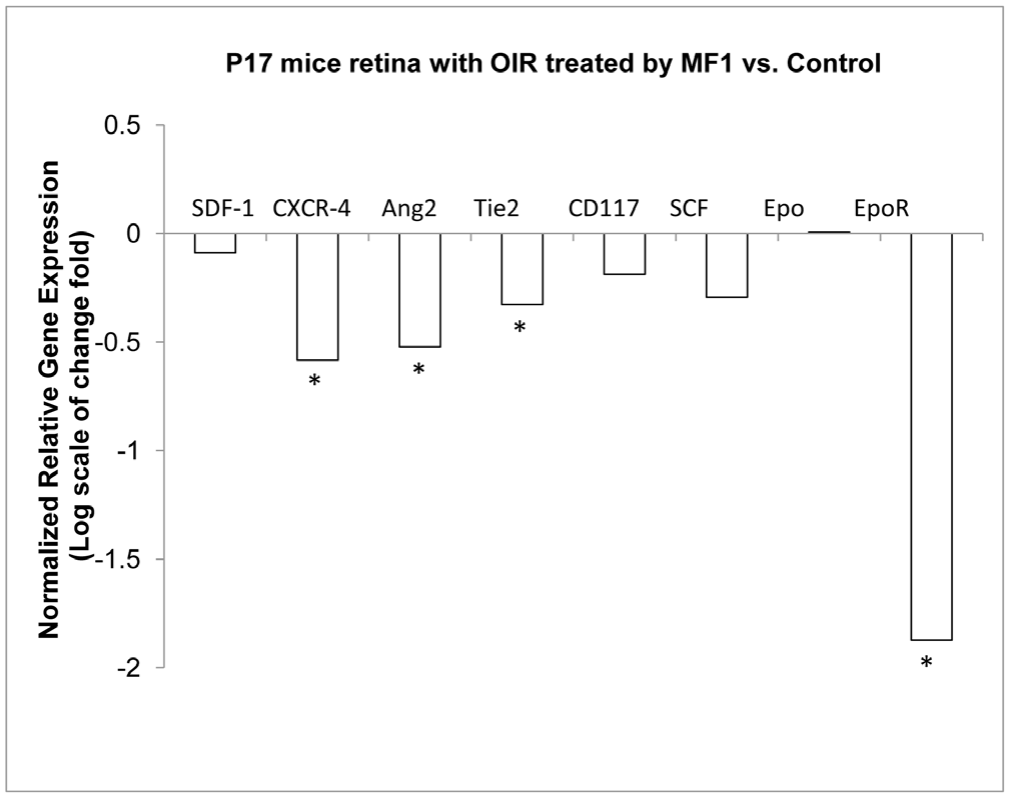
Blockade of VEGFR1 inhibited gene expression of pro-angiogenic factors in OIR. RNA samples were prepared from the retinas of mice that were treated with 25 mg/ml MF1. The results are expressed as normalized relative gene expression or the log(2) scale of mean change fold over control from 5 mice. SDF-1: stromal-derived growth factor-1; CXCR4 (CD184): C-X-C chemokine receptor type 4; Ang2: angiopoietin 2; CD117 (c-kit): mast/stem cell growth factor receptor; SCF: stem cell factor; Epo: erythropoietin; EpoR:erythropoietin receptor. *: p values were less than 0.5 vs. control.

To evaluate the role of VEGFR1 and 2 in ischemia-induced retinal NV, MF1 and DC101 were used to treat mice with OIR at P12 and P15. The dosages evaluated were the same as those for CNV. MF1: 12.5 mg/kg, 25 mg/kg, and 50 mg/kg, DC101: 50 mg/kg, and MF1+DC101: 25 mg/kg each. Both MF1 and DC101 suppressed retinal NV in the OIR model compared to controls and MF1 did so in a dose-dependent manner. At the 50 mg/kg dose, both MF1 and DC101 significantly suppressed retinal NV: DC101 suppressed retinal NV by 54±8% (p = 0.013) and MF1 suppressed retinal NV by 50±7% (p<0.0002); a combination of MF1 and DC101 (25 mg/kg each) had a 62±7% inhibitory efficacy), but without a significant difference compared to MF1 or DC101 treatment alone (Fig. 5). Furthermore, to study the molecular mechanisms by which VEGFR1 signaling regulates pathological angiogenesis in OIR, 4 genes and their respective cognate receptors were selected for measurement of gene expression because they are known to be involved in this model: stromal-derived growth factor-1 (SDF-1)/C-X-C chemokine receptor type 4 (CXCR4) [45]; angiopoietin 2 (Ang2)/(Tie2) [46]; mast/stem cell growth factor receptor (CD117 or c-kit)/stem cell factor SCF) [47]; and erythropoietin (EPO)/erythropoietin receptor (EpoR) [48]. The results demonstrated that blockade of VEGFR1 inhibited the expression of some of these genes to various degrees. Significant reductions for angiogenic factors were as follows: 33±13% reduction for CXCR-4, 30±10% reduction for Ang2, 20±0.7% reduction forTie2, and 73±1.7% reduction for EpoR (Fig. 6).

### Blockade of VEGFR1 and 2 suppressed ischemia-induced vascular leakage in OIR (Fig. 7)

**Figure 7:**
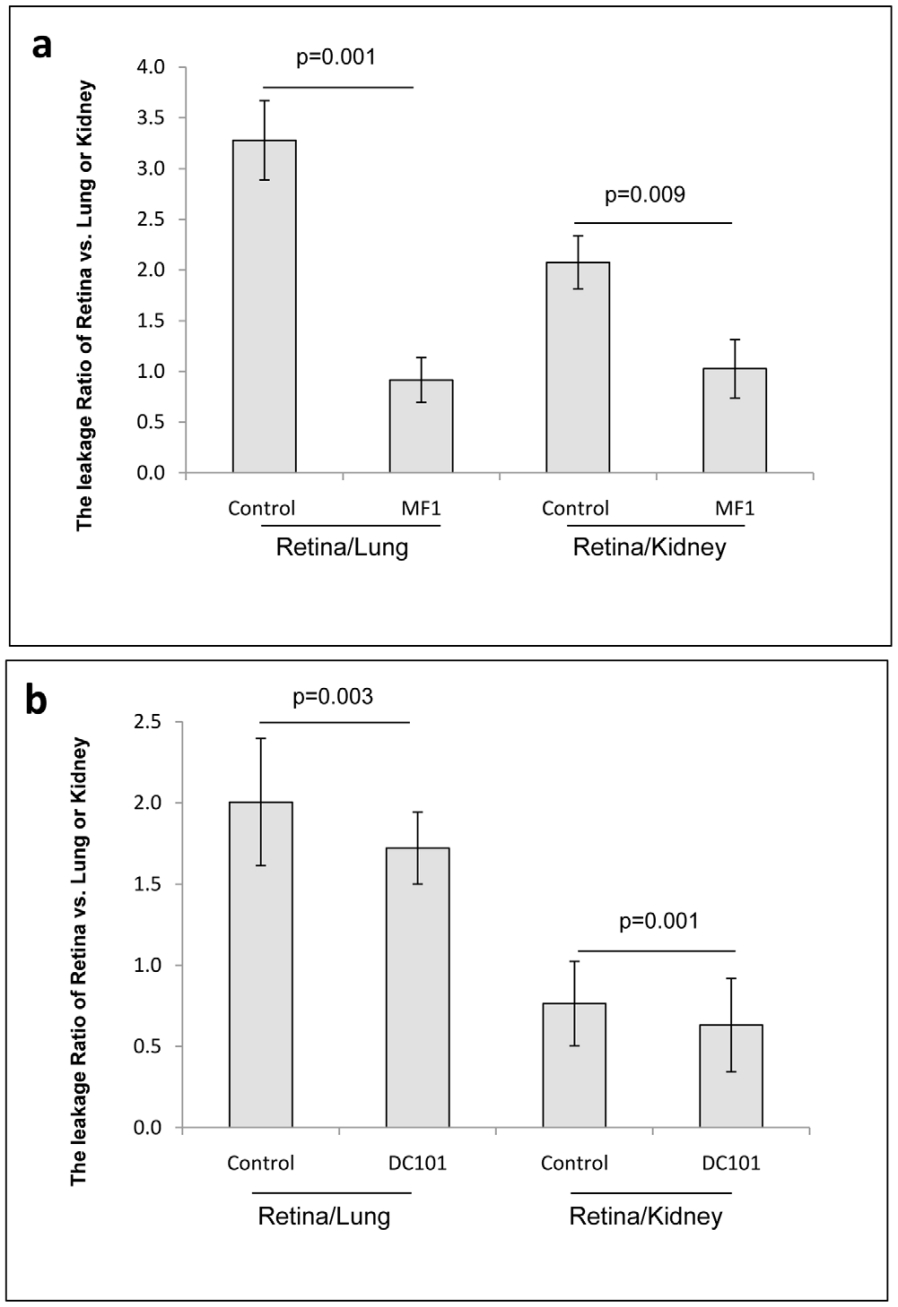
Blockade of VEGFR1 and 2 suppressed vascular leakage in OIR. (a) The leakage ratio of retina to lung or kidney for control and MF1-treated mice with OIR. (b) The leakage ratio of retina to lung or kidney for control and DC101-treated mice with OIR. The BRB assay was performed at P17. The results were expressed as mean±SD of 6–14 individual mice samples.

The dosing and treatment schedules for MF1 and DC101 were the same as described above and the vascular leakage was quantified by measuring [^3^H]-mannitol leakage from blood vessels into the retina as described in Methods. The results showed that MF1 was more effective at inhibiting ischemia-induced vascular leakage due to BRB breakdown than DC101 at the 50 mg/kg dosage (retina to lung leakage ratio for MF1:73±24% inhibition, p = 0.001; for DC101:12±4% inhibition, p = 0.003; retina to renal leakage ratio for MF1:52±28% inhibition, p = 0.009; for DC101:13±4% inhibition, p = 0.001) (Fig. 7).

## Discussion

Previous reports have demonstrated that both VEGFR1 and 2 play very important roles in mediating adverse complications of ischemic and inflammatory disorders, particularly in cancers [25]–[28], and our present study gave further insight into their respective roles and mechanisms in pathological angiogenesis and vascular leakage with the two widely-used mouse models of ocular angiogenesis: laser-induced CNV and OIR. Our observations in this study included (i) consistent with earlier findings [25]–[28], VEGFR1 is involved in pathological angiogenic processes in ischemic retina and targeting VEGFR1 is a potential therapeutic treatment strategy for ischemic retinopathies such as AMD and ROP, (ii) both VEGFR1 and 2 are involved in the angiogenic process through differential mechanisms; combined administration of MF1 and DC101 had additive effects on pathological angiogenesis and vascular leakage, suggesting that combination therapy targeting both receptors would have better efficiency, and (iii) in addition to its role in promoting inflammation associated with ischemia, VEGFR1 signaling is likely involved in the regulation of ischemia-induced BRB breakdown, which makes it an attractive target for the treatment of macular edema. For future prospective therapy, additional studies should be conducted to address the following questions. Is the intraocular administration, which is local and requires less antibody, but is more invasive, and is the widely-used approach for drug delivery in eye diseases, more effective, compared to systemic delivery? What are the safety profiles? Are the two antagonists, MF1 and DC101, effective in other pre-clinical models? Answers to these questions could reveal the potential of VEGF receptor antibodies as therapeutic agents for vasoproliferative ocular disorders.

**Figure 8:**
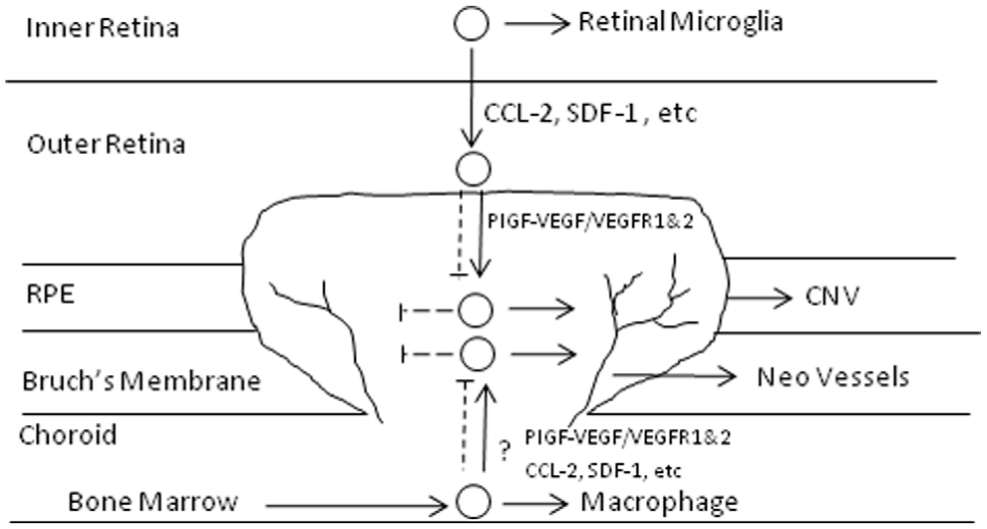
The hypothesized mecanisms regulating recuritment of retinal microgila to CNV. The inflammatory cells in CNV could be recurited retinal microgila, which settle in the inner retina under normal conditions or macrophages from bone marrow that access CNV lesions via blood flow through the choroid. Laser injury up-regulated expression of PlGF, VEGF-A and –B, and VEGFR1 and 2 and could create gradients of some chemotractant factors such as CCL-2 and SDF-1, etc, with higher concentrations in the outer reitna and lower concentrations in the inner retina. The retinal microglial migration towards the CNV was driven by these chemotractant singaling molecues and their further infiltration and activation in CNV are dependent on PlGF-VEGF/VEGFR1 and 2 signaling systems. The exact mechanms controling the recruitment of macrophages is unclear. Line: activation; dished lines: blockade or inhibition.

Inflammation is one of the important mechanisms that promotes CNV formation and two immune responses are associated with its pathogenesis. One is the translocation of resident microglia from the inner to the outer retina and the other is the mobilization and homing of bone marrow-derived leukocytes, such as macrophages [49], [50]. Regardless of the sources, these inflammatory cells, which, once they are recruited into the CNV lesion, are positive for commonly used biomarkers like CD45 and F4/80 and they contribute to CNV formation by secreting cytokines such as TNFα and VEGF. Interestingly, we observed that blockade of VEGFR1 and 2 resulted in the accumulation of inflammatory cells (Lectin^+^CD45^+^ F4/80^+^) in the periphery of the CNV lesions, close to the RPE/retina interface (Fig. 4). Griffonia simplicifolia isolectin-B4 staining is not only a marker for vascular endothelial cells, but it has been identified as a marker for microglia [51], [52]. It would be reasonable that these cells were classified as retinal microglia that migrated from the inner retina, rather than macrophages that were mobilized from bone marrow and the lectin positivity supports this contention. This phenomenon suggested that recruitment of retinal microglia from the inner retina to areas of CNV is separated into two distinct steps, which may be dependent on different signaling molecules. The first is the migration of retinal microglia to the CNV surface, which is independent of PlGF-VEGF/VEGFR1 and 2 signaling, but driven by the gradients of other chemoattractant signal molecules, such as CCL-2/CCR-2 and SDF-1/CXCR4 [53], [54]; the second is the penetration or infiltration and activation of microglia within CNV lesions, which is dependent on PlGF-VEGF/VEGFR1 and 2 signaling [28]. We are not certain of the reasons why two distinct signaling pathways would be required for this process, but it is likely that the phenomenon results from geographic differences of these signal molecules: CCL-2 diffuses into the surrounding environments but PlGF/VEGF is more restricted within CNV, which is necessary for growth of new blood vessels. Circulating macrophages have been reported to play a role in the development of CNV [55]–[57], but it is not clear whether “the two-step” mechanism is implemented for the recruitment of bone-marrow derived leukocytes to CNV. Currently, we don't have evidence to support the possibility and additional studies will be needed to elucidate that. The present results are not designed to dispute the involvement of circulating macrophages in CNV, but to demonstrate the involvement of retinal microglia. The hypothesized mechanisms regulating recruitment of retinal microglia to CNV is shown in Fig. 8.

To quantitatively examine gene expression during CNV pathogenesis, we generated 10–14 CNV (average 12) lesions per eye, which is 9 more than the widely-used mouse model of laser-induced CNV (typically 3 CNV lesions in each eye), and separated the retina and sclera-choroid-RPE. The expression of VEGFR1 and VEGFR2 was increased during CNV development: not at early (3 days after lasering), but late stages (7 and 14 days after lasering). Interestingly, the up-regulation of the two receptors was more pronounced in retina than in choroid at days 7 and 14 (Fig. 1), which might reflect the fact that the CNV outgrows through Bruch membrane and the RPE and invades the retina at the advanced stages, leading to their bundling together with the retina, instead of being limited to the sclera-choroid-RPE portion at the later stage. PlGF showed a consistent increase at all three stages (3, 7 and 14 days after lasering), but VEGF-B only showed a transient up-regulation in choroid at the early stage (3 days after lasering). Despite these relevant findings obtained from the quantitative analysis of gene expression, we recognized that some limitations and potential artifacts may exist in these approaches. First, the fold change of gene expression in the laser treatment groups was not substantial, but only showed moderate increases (from 1.4 to 3.9 fold). Second, expression of VEGF, in contrast to predications, was not significantly up-regulated, but even down-regulated at some points (i.e., in choroid at 14 days). Changes in gene expression in areas of CNV may be difficult to detect since the lesions have a limited pathological area (approximately 200∼300-micron in diameters at 14 days after lasering) compared to the total area of the retina or the sclera-choroid-RPE. The total RNA prepared was under-representative for CNV and the assay may not be sensitive enough for detection of the changes. To overcome these limitations, the isolation of targeted or precise CNV areas, such as by Laser Capture Microdissection (LCM), would be a more preferable approach to quantify the gene expression in this model, which will lead to more significant results.

As stated in the Introduction, the discovery of novel targets for anti-angiogenic therapy, in addition to VEGF, is highly desired and the targeting therapy against VEGFR1 and its ligand PlGF, which can become a potential therapeutic alternative or supplement to anti-VEGF therapy, has drawn increasing attention. Several additional strategies should be explored to overcome the limitations of anti-VEGF therapy [see Introduction, 7–21]. The first strategy would be to target molecules other than VEGF itself. These potential targets include the upstream molecules that regulate VEGF expression (i.e., prolyl hydroxylases and HIFs), the downstream molecules that transmit VEGF signaling (i.e., VEGFR1 and 2 and their receptor kinases) and are regulated by VEGF, such as MEF-2C [58]. Also, the molecules that are involved in maintaining the homeostasis of surrounding or regulatory cells of endothelial cells, such as pericytes/smooth muscle cells, macrophages, astrocytes and Müller cells, or even multiple cell types, are potential targets. Examples of this would be the Angiopoietin/Tie-2 signaling system [59], adhesion molecules such as integrins [60], inflammatory cytokines like TNFα [50], and members of the PDGF family [19], etc. Second, given that angiogenesis is a complex process involving multiple cell types and signaling pathways, it would be more efficient to target multiple molecules or pathways simultaneously rather than one individual molecule or signaling pathway. This could be achieved by effective molecules/compounds, which can target multiple signaling pathways (i.e., TKRs signaling) [61] or a defined combination therapy, which targets two or more specific target molecules or signaling pathways [62]. The third strategy would be to develop more efficient agents (i.e., VEGF-Trap, which blocks all the VEGF isoforms) and capsulize the drugs by sustained delivery devices with the capability of superior-penetrating and slower-releasing features, such as nano-particles. Last, since the ischemic tissues are starving for vessels to supply oxygen and nutrients, which very often leads to the formation of abnormal vasculatures or NV, the inhibition or regression of NV by anti-angiogenic factors has a potential risk of stroke, myocardial infarction, and neuronal toxicity, etc. [63]–[65]. Therefore, a safe means of preventing or reversing pathological NV and BRB breakdown or normalizing pathological NV into functional vasculatures with normal perfusion and barrier function would be desirable for anti-angiogenic therapy. Manipulating the signaling molecules or pathways that regulate stabilization of vasculatures and/or normalization of NV, such as Dll4/Notch signaling, which prevents tip cell formation and branching and was shown to normalize neo vessels in cancer [66], or master transcription factors, such as HIF1α, which controls the expression of VEGF and other genes that regulate vascular biology, metabolism, angiogenesis, proliferation, and survival, would be possible treatment options [67]. The present study has demonstrated that both VEGFR1 and R2 are implicated in pathological angiogenesis and BRB breakdown in ocular disease models and has shown that blocking both receptors is superior to blocking only one. This approach, alone or in combination with targeting other molecules or signaling pathways, could provide greater benefit than currently implemented therapies.
